# Extreme lateral supracerebellar infratentorial approach for tentorial arteriovenous fistula associated with a giant venous ectasia: how I do it

**DOI:** 10.1007/s00701-021-04945-6

**Published:** 2021-08-13

**Authors:** Kyriakos Papadimitriou, Amani Belouaer, Daniele Starnoni, Roy Thomas Daniel

**Affiliations:** Department of Clinical Neurosciences, Department of Neurosurgery, Lausanne University Hospital and University of Lausanne, Rue du Bugnon 46, CH-1011 Lausanne, Switzerland

**Keywords:** Incisural space, Tentorial arteriovenous fistula

## Abstract

**Background:**

Surgery of tentorial dural arteriovenous fistulas (DAVF) associated with large ectatic vein remains challenging due to the intimate neurovascular relationships in the incisural space. Interruption of the arterialized vein requires a good knowledge of the regional anatomy and a precise preoperative evaluation.

**Methods:**

We describe the key steps extreme lateral supracerebellar infratentorial (ELSI) approach for tentorial DAVF with a video illustration. The surgical anatomy is described along with the advantages and limitations of this approach.

**Conclusions:**

In cases of tentorial DAVF where the foot of the arterialized vein is located in the infratentorial compartment, ELSI offers good surgical exposure and outcomes.

**Supplementary Information:**

The online version contains supplementary material available at 10.1007/s00701-021-04945-6.

## Relevant surgical anatomy

The extreme lateral supracerebellar infratentorial (ELSI) is an excellent approach to access the middle and posterolateral incisural space, which corresponds to the ambient and posterolateral aspect of the quadrigeminal cisterns [9]. The roof of this space is formed by the splenium and crus of the fornix medially, the pulvinar and posterior parahippocampal gyrus laterally, and the undersurface of the tentorium. The floor is formed by the culmen of the vermis and quadrangular lobe of the cerebellar hemisphere [7]. Laterally, the middle incisural space extends to the cerebellomesencephalic fissure above the origin of the trigeminal nerve. The crural and ambient cisterns contain major vascular structures namely the anterior choroidal, posterior cerebral artery (PCA), superior cerebellar artery (SCA), and the basal vein of Rosenthal which course around the brainstem parallel to the free edge of the tentorium [7]. Along the approach, the superior petrosal vein is found in an intimate relationship with the CNV. It is formed by the transverse pontine, cerebellopontine fissure, middle cerebellar peduncle, and ponto-trigeminal veins and it drains into the superior petrosal sinus. The trochlear nerve arises just infero-laterally to inferior colliculus and runs anterolaterally in the cerebellomesencephalic fissure towards the middle incisural space to enter its dural canal in the free edge of the tentorium [7]. DAVFs in this area are usually supplied by the marginal tentorial artery (MTA or artery of Bernasconi and Cassinari) [2]. The MTA is a branch of the meningohypophyseal trunk of the internal carotid artery [7].

## Description of the technique (video)

**Fig. 1:**
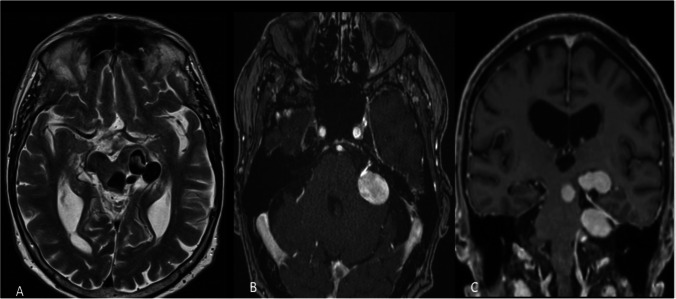
A 76-year-old male presented 2 years ago to another institution with signs and symptoms of intracranial hypertension. On neurological examination, the patient presented a right-side mild hemiparesis, gait disturbance, mild fluent aphasia, and short-term memory problems. Brain imaging revealed an obstructive hydrocephalus due to aqueduct compression in the context of a giant venous ectasia associated with DAVF, Borden type II [1]. The arterial feeders of the DAVF were from the branches of the middle meningeal and occipital artery as well as the artery of Bernasconi-Cassinari. The patient underwent an endoscopic third ventriculostomy and embolization of the arterial afferents. The symptoms of raised intracranial tension resolved but gait instability and memory problems persisted. He was then evaluated at our institute. The MRI showed persistence of the tentorial DAVF associated with multiple giant venous ectasias The MR images showed the major compressive effect on the brainstem associated with intrinsic brainstem hyperintensities on T2W images (Fig. 1A). The DAVF with the foot of the draining vein emerging at the anterior petrotentorial junction was well seen on axial Gd-enhanced T1W MRI (Fig. 1B). The coronal Gd-enhanced T1W MR images (Fig. 1C) showed the tortuous trajectory of the fistulous intradural part of the DAVF in both the infra- and supratentorial compartments along with the compression of the brainstem and mesial temporal lobe

**Fig. 2:**
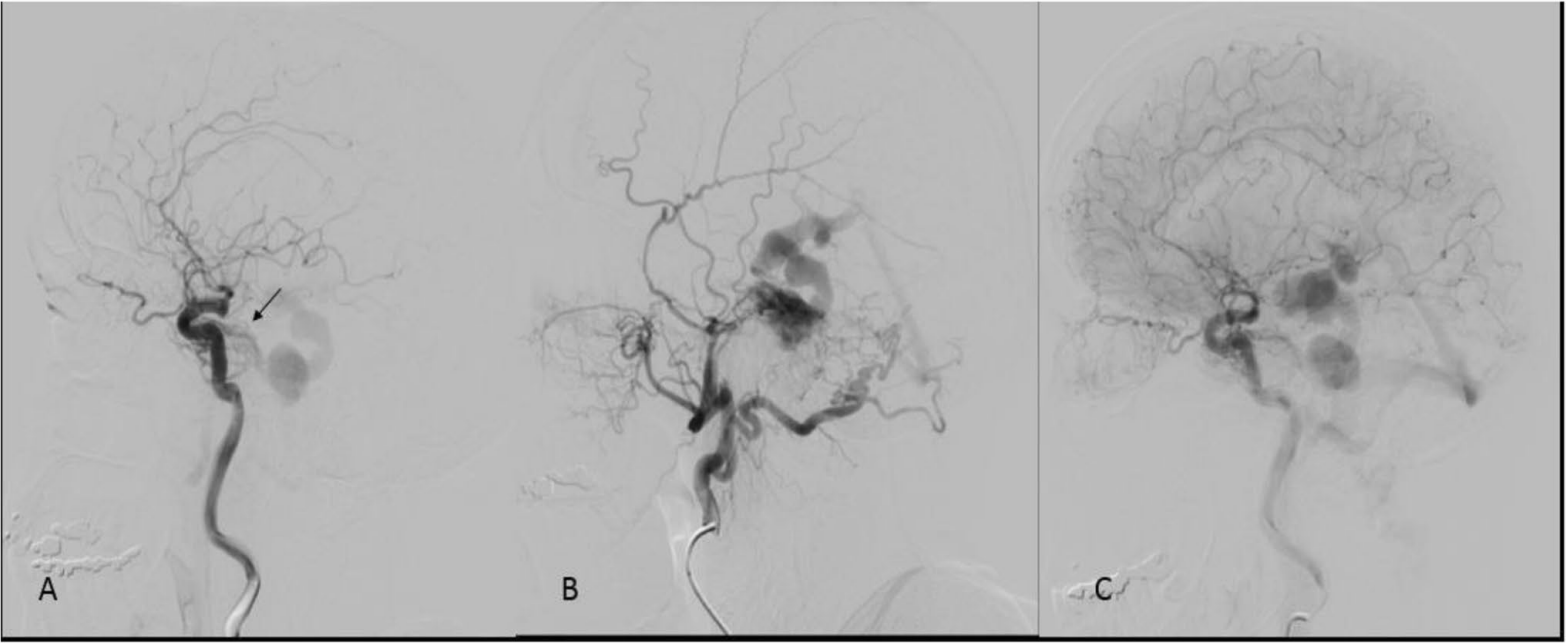
Early arterial phase of the preoperative DSA in the lateral left projection of the ICA injection showed the afferent branches from the artery of Bernasconi-Cassinari (arrow) and early filling of the emergent efferent vein (Fig. 2A). The left external carotid DSA showed several afferent branches from the left occipital and middle meningeal arteries (Fig. 2B). The DSA in the venous phase demonstrated the multiple giant venous ectasias (Fig. 2C)

**Fig. 3:**
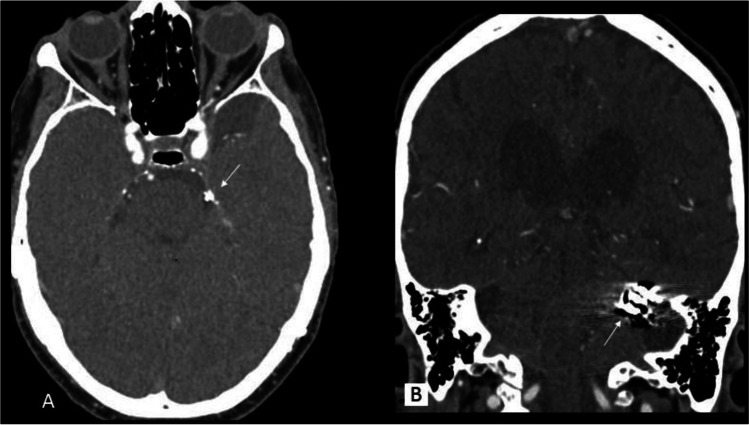
Postoperative angio-CT scan showed the clip (arrow) of the DAVF in position and disappearance of the fistulous connection. The brainstem and left mesial temporal lobe regained their normal positions in the middle incisural space and middle fossa, respectively (axial and contrast enhanced coronal CT images). MRI was not performed due to possible clip artifacts

**Fig. 4:**
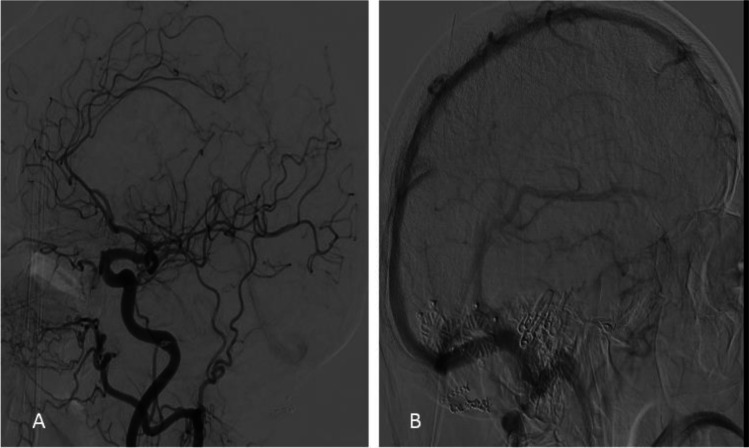
Postoperative DSA (lateral projection) performed 1 year following surgery confirmed the disappearance of the fistulous connection and the tortuous venous ectasias ectatic (common carotid injection with arterial phase in Fig. 4A and venous phase in Fig. 4B). At this follow-up visit, the patient had no gait instability and the memory functions had significantly improved. There were no symptoms of hydrocephalus and raised intracranial tension

The patient is placed in a lateral park-bench position, with the head in maximal flexion and the bed raised 20° above the horizontal to facilitate retraction of the cerebellum. Continuous neuro-monitoring of cranial nerves IV to XI is performed. A curvilinear retroauricular incision extending from the mastoid tip to the posterior temporal region is performed. A craniotomy allows the exposition of the lateral part of the transverse sinus. Transverse sigmoid junction and upper sigmoid sinus allow adequate access for this approach. Repair of any opened mastoid air cells is essential. A 5- to 8-mm durotomy parallel to the transverse and sigmoid sinuses is performed. Dural hitch stitches allow mobilization of the sinuses to improve vision. The lateral cerebellomedullary cistern is opened to drain CSF and relax the cerebellum. The surgical corridor is situated between the inferior surface of the tentorium and the quadrangular lobule. A more inferior and medial retraction of the quadrangular lobule of the cerebellum can expand the exposure of the cerebellomesencephalic fissure and the cerebellopontine cistern. This lateral cerebellar corridor, combined with the supracerebellar, expands the access to the infratentorial part of the middle incisural space allowing the surgeon to work in two windows, on either side of the superior petrosal vein and the trigeminal nerve. The exploration of the cerebellomesencephalic fissure reveals the foot of the fistulous venous communication attached to the DAVF along with the venous aneurysms in the infratentorial compartment producing brainstem compression (Figs. 1–2). The venous ectasia is then carefully dissected and mobilized, in order to have a good exposure of the fistulous arterialized venous connection, which is also confirmed indocyanine green (ICG) video angiography. A clip is then applied and a repeat ICG injection is performed to verify its occlusion. The venous aneurysm needed to be decompressed to allow proper visualization of the infratentorial surface of the DAVF up to the porus trigeminus to ensure all venous connections to the DAVF is excluded. The retrograde flow though venous aneurysm is clipped using large curved clips (Figs. 3–4). The dura is closed watertight and the bone flap replaced. The surgical wound is closed in layers.

## Indications

Tentorial DAVFs represent a rare subgroup and accounts up to 4% of all DAVFs [3]. They present usually an aggressive behavior, with inherent risk of hemorrhage and progressive focal neurological deficits [4]. DAVFs with retrograde filling of leptomeningeal veins present a high risk of morbidity and mortality. Trans-arterial exclusion of feeders is not an effective and durable treatment. In case of direct non-sinus-type DAVF such as tentorial DAVF, interruption of the arterialized vein near the dural entry/exit point with clip application or anatomical disconnection without obliteration of the meningeal feeding arteries and/or excising the arterialized dura is associated with satisfactory surgical outcomes [10]. However, with trans-arterial embolization, occlusion rates are significantly lower. Of note, transvenous endovascular routes are rarely utilized due to the high risk of rupture of an already fragile arterialized vein. In cases of tentorial DAVF where the foot of the arterialized vein is located in the infratentorial compartment, ELSI offers an excellent surgical exposure.

## Limitations

The ELSI implies long working distances that necessitates microsurgical skills with a steep learning curve [6]. It requires special attention in dealing with venous sinuses and harbors a risk of sinus thrombosis.

## How to avoid complications

Sinuses skeletonization should be carefully performed in order to avoid sinus injury. Thereafter, the sinuses need to be covered with a wet Gelfoam in order to be protected from any thermal injury from the operating microscope. Excessive traction of the sinuses should be avoided to prevent any mechanical injury. Release of CSF from the cisterns helps in reducing excessive cerebellar retraction.

## Preoperative workup

Precise preoperative planning with special consideration to the location of the foot of the draining vein of the DAVF is imperative. Preoperative brain MRI and computed tomography (CT) scan with bone windows are performed to study the DAVF anatomy and its relationships to adjacent structures [4]. Digital subtraction angiography (DSA) is essential to evaluate the afferent arterial supply as well as the anatomy of the efferent draining vein [5, 8].

## Postoperative workup

Postoperative DSA is performed to verify complete disconnection of the draining vein. In case of perioperative bleeding, a brain CT is performed in order to monitor the ventricular size. MRI is performed at 3 months follow-up to document regression in size of the venous aneurysms.

## Instructions for the postoperative care

The patient is hospitalized in the intermediate care unit for 24 h. Neurological examination is performed in order to detect any potential complications related to surgery. The patient is kept well hydrated to avoid any sinuses thrombosis. Strict blood pressure monitoring is also recommended.

## Specific information to give to the patient about surgery and potential risks

Surgery of DAVF is not without significant morbidity. The most frequent complications are hemorrhage, wound infections, posterior fossa ischemia, hydrocephalus, and new cranial nerve deficit. Failure in locating or complete disconnection of the DAVF draining vein will ensue in persistent AV shunt with inherent risk of rebleeding and/or persistent venous hypertension. Re-permeabilization of the DAVF, though rare, should be kept in mind [5, 8].

## Supplementary Information

Below is the link to the electronic supplementary material.

Supplementary file1 (MP4 162828 KB)
